# Time interval-based indication for liver resection of metastasis from pancreatic cancer

**DOI:** 10.1186/s12957-020-02058-5

**Published:** 2020-11-10

**Authors:** Yusuke Mitsuka, Shintaro Yamazaki, Nao Yoshida, Masahiro Yan, Tokio Higaki, Tadatoshi Takayama

**Affiliations:** Department of Digestive Surgery, Nihon University School of Medicine, 30-1 Ohyaguchikami-machi, Itabashi-ku, Tokyo, 173-8610 Japan

**Keywords:** Pancreatic cancer, Pancreatectomy, Liver resection, Liver metastases, Indication

## Abstract

**Background:**

Surgical indications for liver metastases from pancreatic ductal adenocarcinoma (PDAC) are lacking because outcomes are usually poor. However, liver resection and the recent progress in perioperative chemotherapy have been observed to improve survival.

**Methods:**

We performed liver resection for liver metastases from PDAC only under the following criteria: (1) liver-only metastasis, (2) up to three tumors, and (3) no increase in the number of metastases during the 3-month observation period. No limitations were placed on the location or size of liver metastasis. In this study, we aimed to validate our surgical criteria and analyze factors affecting survival in patients with PDAC.

**Results:**

Seventy-nine patients underwent curative resection for PDAC between 2005 and 2015. Seventy-one patients experienced recurrence, with liver-only recurrence in 17 patients. Among these, nine patients underwent liver resection and eight did not. The median survival time was significantly better for patients who underwent liver resection (55 months) than for those with other recurrences (17.5 months, *p* = 0.016). The median survival after liver recurrence was significantly better in the liver resection group (31 months) than in the non-liver resection group (7 months, *p* = 0.0008). The median disease-free interval (DFI) after pancreatectomy was significantly longer in the liver resection group (21 months; range, 3–44 months) than in the non-liver resection group (3 months; range, 2–7 months; *p* = 0.02).

**Conclusion:**

Good indications for liver metastases from PDAC include solitary metachronous tumors and longer DFIs.

## Introduction

Pancreatic ductal adenocarcinoma (PDAC) has a high frequency of recurrence even after curative resection [1–3]. The postoperative recurrence rate was 30–50% during the first 2 years, and the overall 5-year survival rate was approximately 20% [4, 5]. Therefore, most patients require a second treatment after pancreatectomy [6]. The median survival time for unresectable patients was 11.1 and 8.5 months with FOLFIRINOX (5-fluorouracil, leucovorin, irinotecan, and oxaliplatin) and GnP (gemcitabine and nab-paclitaxel) as first treatments, respectively [7, 8]. However, performing full-dose systemic chemotherapy was difficult in patients who have undergone pancreatectomy.

Recently, advances in perioperative management have allowed liver resection for non-colorectal cancers [9, 10]. However, deducing the surgical indications for the resection of liver metastases from each type of cancer has been difficult owing to the limited number of participants. Moreover, liver resection after pancreaticoduodenectomy is complex because biliary reconstruction makes hepatic inflow occlusion during liver transection difficult [4, 11, 12]. Therefore, liver resection for PDAC has rarely been studied, and factors affecting survival have been poorly documented.

We have been performing liver resection for metastases from PDAC using our unique criteria over the past decade. Based on our observations, we have compiled this article to highlight the benefits of liver resection for liver-only metastasis from PDAC and validate the effectiveness of our criteria based on indications.

## Patients and methods

### Patients

Between 2005 and 2015, data were collected from patients who underwent pathologically curative resection for PDAC. All patients were followed up every 4 months after pancreatic resection to measure tumor marker levels and underwent to monitor the tumor’s growth using contrast-enhanced computed tomography (CT). When a local or distant metastasis was suspected, magnetic resonance imaging or positron emission tomography-CT was additionally performed every month during follow-up. A recurrence was confirmed when two or more modalities or a recurrent lesion showed visible recurrence at two different time points.

### Indications for liver resection

When metachronous liver metastases were found in up to three tumors, liver resection was considered after a 3-month observation period. Patients were classified into two groups: the liver resection and the non-liver resection groups. Indications for the liver resection group were as follows: (1) liver-only metastasis, (2) up to three tumors, and (3) no increase in the number of metastases during the 3-month observation period. There were no limitations on the location or size of the tumors. When the patient received adjuvant chemotherapy, liver resection was planned after an interval of ≥ 3 weeks from the last chemotherapy. All patients showed Child class A liver function and no contraindications for general anesthesia. The non-liver resection group comprised patients not meeting the above criteria; they received the standard systemic chemotherapy. Indications for a second liver resection were the same as those for the first liver resection. However, a second liver resection for liver-only metastasis was considered only for solitary recurrence after the first liver resection with a disease-free interval (DFI) ≥ 12 months.

### Liver resection

Liver resection was performed using the clamp-crushing method, and perioperative care was carried out according to institutional protocols as described by Yamazaki et al*.* [13]. Non-anatomical partial resection is the first-line procedure for liver metastases. When hepatic inflow occlusion was impossible because of pancreaticoduodenectomy, liver resection was performed without Pringle’s maneuver. The median total follow-up time after pancreatectomy was 55 months (range, 15–119 months).

### Assessment

Patient characteristics were compared between the liver resection and non-liver resection groups for liver-only metastasis. To assess the benefits of liver resection, the overall survival was compared between the liver resection group and all other patients showing recurrence. Finally, to identify the impact of resection in liver-only recurrence, survival times from the time of recurrence were compared between the liver and non-liver resection groups. Complications were ranked using the Clavien-Dindo classification system as severe (grade IIIb or higher). The institutional review board attested all the protocols in this study (registration number RK-150414-4). Informed consent was obtained from all the patients.

### Statistical analysis

Data are expressed as medians and ranges or as absolute values and percentages. Student’s *t* test, the *χ*^2^ test, and Fisher’s exact test were used for univariate analysis. Survival curves were obtained using the Kaplan–Meier method, and distributions were compared using the log-rank test. *P* values < 0.05 were considered indicative of statistical significance. All statistical analyses were performed using the software, JMP Statistical Discovery version 13.2 (SAS Institute, Cary, NC).

## Results

### Pancreatectomy and liver metastases

**Fig. 1:**
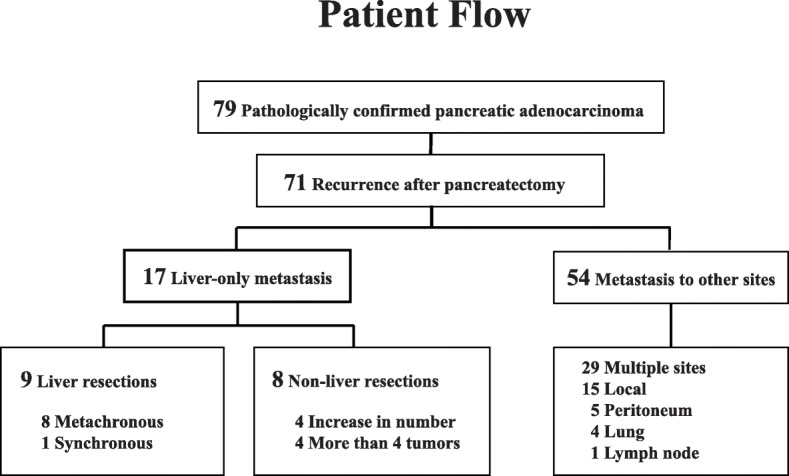
Patient flow chart. Of the 79 patients, 71 experienced recurrence during follow-up, including 17 patients with liver-only metastasis. Among these, nine patients performed liver resection (liver resection group) and eight patients did not undergo liver resection (non-liver resection group)

Between 2005 and 2015, 79 patients underwent pathologically curative resection for PDAC. The baseline characteristics of all patients are shown in Table 1. Twenty patients (25.3%) received adjuvant chemotherapy after pancreatectomy with S-1 or gemcitabine. This study excluded pathologically diagnosed intraductal papillary mucinous carcinoma because its biological recurrence rate differed from that of PDAC. Of the 79 patients in our study, 71 experienced recurrences during follow-up (Fig. 1), including 17 patients with liver-only metastasis. Of the 17, liver resection was performed only for nine patients, the liver resection group, classified based on indications. The remaining eight, the non-liver resection group, did not undergo liver resection; four had ≥ 4 tumors, and four exhibited increasing tumor numbers during the observation period.

**Table 1:** Baseline characteristics of all patients with pancreatic cancer

		(*n* = 79)
Age	(years)	66 (41–83)
Primary operation	(%)	
pancreaticoduodenectomy		50 (63.3)
Distal pancreatectomy		28 (35.4)
Total pancreatectomy		1 (1.3)
TMN classification stage	(%)	
IA		4 (5.1)
IB		6 (7.6)
IIA		24 (30.4)
IIB		18 (22.7)
III		26 (32.9)
IV		1 (1.3)
Serum bilirubin	(IU/L)	0.69 (0.2–11.7)
Serum albumin	(IU/L)	4.0 (3.5–4.9)
Carcinoembryonic antigen	(IU/L)	3.85 (0.6–255)
Carbohydrate antigen 19-9	(IU/L)	104.9 (0.1–13,820)
Operation time	(min)	446 (193–738)
Intraoperative blood loss	(ml)	370 (25–1720)
Adjuvant chemotherapy after pancreatectomy	(%)	20 (25)

Values represent median with range

### Characteristics of patients with liver-only recurrence

Comparing the 17 patients with liver-only metastasis, we observed that the median DFI after pancreatectomy was significantly longer in the liver resection group than in the non-liver resection group (21 months, range [3–44 months]; 3 months, range [2–7 months], respectively; *p* = 0.017) (Table 2). The number of tumors was significantly higher in the non-liver resection group owing to our criteria for resection (based on indications). No other significant differences in patient characteristics were observed. Although liver resection was performed without Pringle’s maneuver in 6 patients with pancreaticoduodenectomy, intraoperative blood loss was minimal (median, 246.5 ml; range, 53–508 ml), and the operation time (median, 313 min; range, 88–413 min) was not extended. No patient had severe complications according to the Clavien-Dindo classification.

**Table 2:** Characteristics of patients with liver-only recurrence

		Liver resection	Non-liver resection	*P* value
		(*n* = 9)	(*n* = 8)	
Age	(years)	66 (41–74)	65 (43–74)	0.97
Primary operation				
Pancreaticoduodenectomy		6	4	
Distal pancreatectomy		3	4	
Albumin	(g/dL)	3.9 (3.5–4.3)	4.05 (2.4–4.5)	0.59
Serum bilirubin	(μmol/L)	0.52 (0.25–4.23)	1.78 (0.33–3.04)	0.05
Prothrombin time (INR)		0.965 (0.92–1.14)	0.965 (0.88–1.03)	0.51
Carcinoembryonic antigen	(IU/L)	5.1 (1.7–15.2)	7.8 (2.8–14.5)	0.21
Carbohydrate antigen 19-9	(IU/L)	148.8 (15.2–13,820)	869 (0.1–5210)	0.99
Disease-free interval from pancreatectomy	(months)	21 (3–44)	3 (2–7)	0.02
Number of liver metastases		1 (1–3)	7 (4–15)	< 0.001
Adjuvant chemotherapy after pancreatectomy	(%)	4 (44.4)	2 (25.0)	0.49
Operation time	(min)	302 (53–413)	N/A	
Intraoperative blood loss	(mL)	186 (53–508)	N/A	
Morbidity (Clavien-Dindo clasification > IIIb)	(%)	1 (11.1)	N/A	
Mortality	(%)	0	N/A	

Values represent median with range

*DFI* Disease-free interval, *N/A* Not applicable

### Survival

**Fig. 2:**
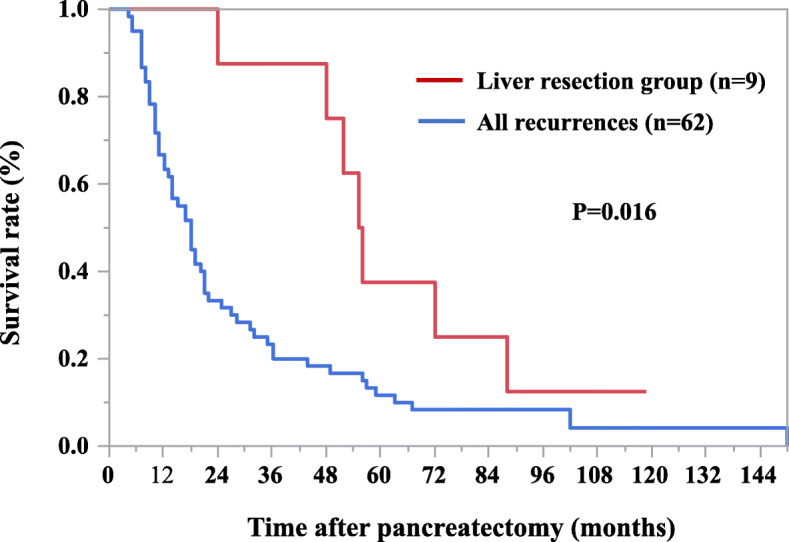
Overall survival for all participants. Median overall survival was significantly better in liver resection group compare to the all patients who had recurrence (55 months vs. 17.5 months, *p* = 0.016)

**Fig. 3:**
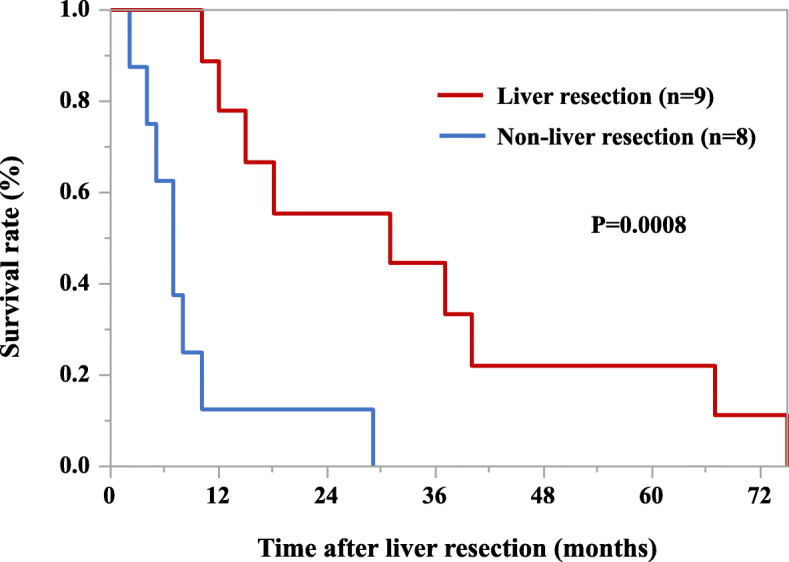
Survival after liver recurrence. Regarding liver-only metastasis, median overall survival is significantly better in the liver resection group (44.0 months) than in the non-liver resection group (19.9 months, *p* = 0.0008).

The median overall survival was significantly better in the liver resection group than in all the other patients who had a recurrence (55 months vs. 17.5 months, *p* = 0.016) (Fig. 2). Regarding liver-only metastasis, the median survival time after liver recurrence was significantly better in the liver resection group than in the non-liver resection group (31 months vs. 7 months, *p* = 0.0008) (Fig. 3). In the liver resection group, the median survival after liver resection was 25 months (range, 10–70 months) (Table 3). Two patients underwent two liver resections based on the same criteria as those defined for the first resection.

**Table 3:** Characteristics of liver resection group

Case	Primary operation	Diagnosis	DFI (months)	Number	Diameter (mm)	Treatment after recurrence	Survival after liver resection (months)	Total follow-up (months)	
1	Pancreaticoduodenectomy	Metachronous	37	1	25	Chemotherapy	10	48	Deceased
2	Distal pancreatectomy	Metachronous	3	1	26	Chemotherapy	39	72	Deceased
3	pancreaticoduodenectomy	Metachronous	21	1	24	Liver resection	67 (24)^a^	88	Deceased
4	Distal pancreatectomy	Metachronous	12	1	24	None	11	24	Deceased
5	pancreaticoduodenectomy	Metachronous	44	1	18	Liver resection	70 (55)^a^	119	Alive
6	Distal pancreatectomy	Metachronous	33	2	36	Chemotherapy	10	51	Deceased
7	pancreaticoduodenectomy	Metachronous	20	3	22	Chemotherapy	33	56	Deceased
8	pancreaticoduodenectomy	Metachronous	25	3	36	Chemotherapy	25	55	Deceased
9	pancreaticoduodenectomy	Synchronous	-	1	8	Chemotherapy	15	15	Deceased

*DFI* Disease-free interval

^a^After second liver resection

## Discussion

This study revealed that patients with up to three metachronous liver-only metastases from PDAC may be good candidates for liver resection. The observation time and DFI were key to optimizing the selection of patients for liver resection.

Regarding the timing of the operation, a constant number of tumors during the 3-month observation period and a long DFI may be indispensable indications. Four of the eight patients in the non-liver resection group presented an increase in the number of liver metastases during the 3-month observation period. However, multiple liver metastases were found during the subsequent 3 months. One patient was found with synchronous liver-only metastasis (discovered incidentally during the operation). Besides, studies in the past have revealed that liver resection for synchronous liver metastasis has not been beneficial to patients [14–16]. Moreover, a significantly longer DFI was observed in the liver resection group than in the non-liver resection group. This may reflect tumor aggressiveness and malignant potential in the primary PDAC [17, 18]. Therefore, a longer DFI appears to be an important factor for improving our selection criteria.

Regarding the participants who underwent the operation, no reports have mentioned long-term survivors after two liver resections from PDAC. In this study, two patients underwent two liver resections and survived for more than 7 years after pancreatectomy. They also had a longer DFI after pancreatectomy and the first liver resection. The indications for the second liver resection were the same as those for the first one. Therefore, our criteria seem mandatory for the optimal selection of patients for a second liver resection.

In general, operations after pancreaticoduodenectomy are complex because the occlusion of blood inflow is difficult. This may result in a higher blood loss during liver transection. In our study, none of the cases showed an overly long operation time or excessive intraoperative blood loss. Recent technical advances in liver resection have allowed safer liver transection in patients with prior pancreaticoduodenectomy [11, 12]. To avoid huge blood loss, we performed total liver mobilization and lifting of the liver transection site to the ventral side when the tumor was located in the right lobe. This method was effective in controlling intraoperative blood loss. Therefore, this study showed that liver resection for PDAC metastasis was reasonably safe.

A limitation of this study, however, was the small sample size attributed to the rarity of the pathological entity. Therefore, this study may include selection bias in terms of treatment. Therefore, further data collection is needed to analyze the effectiveness of liver resection for PDAC metastases.

## Conclusions

Liver resection improves survival in patients with recurrent liver-only metastasis from PDAC. We believe that our criteria for selecting patients for liver resection based on indications work reasonably well.

## Data Availability

All data was collected and kept by the corresponding author. Someone who wants to see its data, the part of data is available with contacting the corresponding author.

## Abbreviations

PDAC: Pancreatic ductal adenocarcinoma
DFI: Disease-free interval
FOLFIRINOX: 5-Fluorouracil, leucovorin, irinotecan, and oxaliplatin
GnP: Gemcitabine and nab-paclitaxel

## References

[1] Gleisner AL, Assumpcao L, Cameron JL, Wolfgang CL, Choti MA, Herman JM (2007). Is resection of periampullary or pancreatic adenocarcinoma with synchronous hepatic metastasis justified?. Cancer..

[2] Klein F, Puhl G, Guckelberger O, Pelzer U, Pullankavumkal JR, Guel S (2012). The impact of simultaneous liver resection for occult liver metastases of pancreatic adenocarcinoma. Gastroenterol Res Pract.

[3] Shrikhande SV, Kleeff J, Reiser C, Weitz J, Hinz U, Esposito I, et al. Pancreatic resection for M1 pancreatic ductal adenocarcinoma. Ann Surg Oncol. 14(1):118–27. 10.1245/s10434-006-9131-8 17066229

[4] Adam R, Chiche L, Aloia T, Elias D, Salmon R, Rivoire M (2006). Hepatic resection for noncolorectal nonendocrine liver metastases: analysis of 1452 patients and development of a prognostic model. Ann Surg.

[5] Yeo CJ, Cameron JL, Lillemoe KD, Sitzmann JV, Hruban RH, Goodman SN (1995). Pancreaticoduodenectomy for cancer of the head of the pancreas. 201 patients. Ann Surg.

[6] Crippa S, Bittoni A, Sebastiani E, Partelli S, Zanon S, Lanese A (2016). Is there a role for surgical resection in patients with pancreatic cancer with liver metastases responding to chemotherapy?. Eur J Surg Oncol.

[7] Conroy T, Desseigne F, Ychou M, Bouché O, Guimbaud R, Bécouarn Y, Groupe Tumeurs Digestives of Unicancer, PRODIGE Intergroup (2011). FOLFIRINOX versus gemcitabine for metastatic pancreatic cancer. N Engl J Med.

[8] Von Hoff DD, Ervin T, Arena FP, Chiorean EG, Infante J, Moore M (2013). Increased survival in pancreatic cancer with nab-paclitaxel plus gemcitabine. N Engl J Med.

[9] Metcalfe MS, Mullin EJ, Maddern GJ (2006). Hepatectomy for metastatic noncolorectal gastrointestinal, breast and testicular tumours. ANZ J Surg.

[10] Groeschl RT, Nachmany I, Steel JL, Reddy SK, Glazer ES, C de Jong MC (2012). Hepatectomy for noncolorectal non-neuroendocrine metastatic cancer: a multi-institutional analysis. J Am Coll Surg.

[11] De Jong MC, Farnell MB, Sclabas G, Cunningham SC, Cameron JL, Geschwind JF (2010). Liver-directed therapy for hepatic metastases in patients undergoing pancreaticoduodenectomy: a dual-center analysis. Ann Surg.

[12] Hartwig W, Hackert T, Hinz U, Hassenpflug M, Strobel O, Büchler MW (2009). Multivisceral resection for pancreatic malignancies: risk-analysis and long-term outcome. Ann Surg.

[13] Yamazaki S, Takayama T, Moriguchi M, Mitsuka Y, Okada S, Midorikawa Y (2012). Criteria for drain removal following liver resection. Br J Surg.

[14] Michalski CW, Erkan M, Huser N, Müller MW, Hartel M, Friess H (2008). Resection of primary pancreatic cancer and liver metastasis: a systematic review. Dig Surg.

[15] Singh A, Singh T, Chaudhary A (2010). Synchronous resection of solitary liver metastases with pancreaticoduodenectomy. JOP..

[16] Takada T, Yasuda H, Amano H, Yoshida M, Uchida T (1997). Simultaneous hepatic resection with pancreato-duodenectomy for metastatic pancreatic head carcinoma: does it improve survival?. Hepatogastroenterology..

[17] Spinelli GP, Zullo A, Romiti A, Di Seri M, Tomao F, Miele E (2006). Long-term survival in metastatic pancreatic cancer. A case report and review of the literature. JOP..

[18] Adham M, Jaeck D, Le Borgne J, Oussoultzouglou E, Chenard-Neu MP, Mosnier JF (2008). Long-term survival (5-20 years) after pancreatectomy for pancreatic ductal adenocarcinoma: a series of 30 patients collected from 3 institutions. Pancreas..

